# Selective function-blocking monoclonal human antibody highlights the important role of membrane type-1 matrix metalloproteinase (MT1-MMP) in metastasis

**DOI:** 10.18632/oncotarget.13157

**Published:** 2016-07-11

**Authors:** Albert G Remacle, Piotr Cieplak, Dong Nam Hyun, Sergey A Shiryaev, Xin Ge, Alex Y Strongin

**Affiliations:** ^1^ Infectious and Inflammatory Disease Center/Cancer Research Center, Sanford Burnham Prebys Medical Discovery Institute, La Jolla, CA 92037, USA; ^2^ Department of Chemical and Environmental Engineering, University of California, Riverside, Riverside, CA 92512, USA

**Keywords:** metastasis, cancer, MT1-MMP, antibody, proteinase

## Abstract

The invasion-promoting MT1-MMP is a cell surface-associated collagenase with a plethora of critical cellular functions. There is a consensus that MT1-MMP is a key protease in aberrant pericellular proteolysis in migrating cancer cells and, accordingly, a promising drug target. Because of high homology in the MMP family and a limited success in the design of selective small-molecule inhibitors, it became evident that the inhibitor specificity is required for selective and successful MT1-MMP therapies. Using the human Fab antibody library (over 1.25×10^9^ individual variants) that exhibited the extended, 23-27 residue long, V_H_ CDR-H3 segments, we isolated a panel of the inhibitory antibody fragments, from which the 3A2 Fab outperformed others as a specific and potent, low nanomolar range, inhibitor of MT1-MMP. Here, we report the in-depth characterization of the 3A2 antibody. Our multiple *in vitro* and cell-based tests and assays, and extensive structural modeling of the antibody/protease interactions suggest that the antibody epitope involves the residues proximal to the protease catalytic site and that, in contrast with tissue inhibitor-2 of MMPs (TIMP-2), the 3A2 Fab inactivates the protease functionality by binding to the catalytic domain outside the active site cavity. In agreement with the studies in metastasis by others, our animal studies in acute pulmonary melanoma metastasis support a key role of MT1-MMP in metastatic process. Conversely, the selective anti-MT1-MMP monotherapy significantly alleviated melanoma metastatic burden. It is likely that further affinity maturation of the 3A2 Fab will result in the lead inhibitor and a proof-of-concept for MT1-MMP targeting in metastatic cancers.

## INTRODUCTION

Metastatic spread is frequently lethal to cancer patients and the treatment options are frequently limited. In multiple cancer types, the level of zinc-dependent matrix metalloproteinases (MMPs) is increased to allow metastatic cells to degrade the extracellular matrix and to invade the tissue and distant organs [1-5].

There are 23 individual MMPs in humans from which 18 proteinases are soluble and 6 are membrane-tethered [membrane type (MT)-MMPs] [6]. Soluble MMP proenzymes typically contain an N-terminal inhibitory prodomain followed by a catalytic domain (CAT), a flexible hinge linker and a hemopexin domain [7]. In addition, MT-MMPs also includes either a transmembrane domain followed by a cytoplasmic tail domain (MT1-, MT2-, MT3- and MT5-MMP) or a glycosylphosphatidylinositol moiety (MT4- and MT6-MMP) that tethers these proteases to the cell membrane [7]. MMPs are zinc-dependent proteinases and they share the active site zinc binding motif HEXXHXXGXXH in which the His residues coordinate the catalytic zinc ion [8]. MMPs are synthesized as latent zymogens that require proteolytic activation to become functional proteases. In this process, the N-terminal inhibitory prodomain is removed and the catalytic site of the emerging mature enzyme becomes liberated and exposed. It is established that pro-metastatic, collagenolytic membrane-tethered MT1-MMP/MMP-14 functions as a main mediator of the pro-migratory proteolytic events at the cell surface [9, 10]. Expression of MT1-MMP directly correlates with poor clinical outcome, blood vessel invasion and high incidence of distant metastasis in multiple cancer types [11-13]. MT1-MMP null mice are dwarfs with skeletal abnormalities and soft tissue disorders, and they die prior to adulthood, thus supporting the crucial function of MT1-MMP in cell migration during gastrulation and collagen turnover [14]. Overall, there is a consensus among researchers that because of its importance in promoting cell invasion and metastasis MT1-MMP is a promising drug target in cancer and certain other pathologies [11-13, 15-19].

MT1-MMP is regulated both as a protease and as a membrane-anchored protein. Similar with other MMPs, the proteolytic removal of the inhibitory prodomain is required for the conversion of the latent, 63 kDa, MT1-MMP zymogen into the functionally active, 55 kDa, protease. The furin-like proprotein convertase activity is essential to the prodomain removal and MT1-MMP activation [20-22]. Once activated, MT1-MMP can be efficiently inhibited by its natural protein inhibitors, tissue inhibitors of MMPs (TIMPs) [23]. The binding of TIMPs to the MT1-MMP active enzyme results in the stoichiometric and proteolytically inactive TIMP·MT1-MMP complex. There are four individual TIMPs in humans (TIMP-1, -2, -3, and -4) [23, 24]. With the exception of TIMP-1, TIMPs are efficient, sub-nanomolar inhibitors of MT1-MMP [25, 26]. The MT1-MMP/TIMP balance is arguably the most significant factor in the regulation of the net proteolytic activity of cellular MT1-MMP. As a membrane-tethered protease, MT1-MMP is also regulated *via* cellular compartment trafficking, internalization and recycling [4, 27, 28]. These coordinated, multi-dimensional mechanisms regulate MT1-MMP spatially and temporally, and they concentrate the MT1-MMP activity on the leading and trailing edges in migrating cells [10].

Through earlier trial and error, it became evident that the inhibitor specificity is required for selective and successful MMP therapies [29-33]. Accomplishing the required target specificity and selectivity with small-molecule MMP inhibitors is exceedingly difficult and so far the success has been limited. Because the catalytic mechanism and the catalytic domain fold are largely conserved in the MMP family members, the small-molecule inhibitors simultaneously interact with multiple MMPs resulting in off-target effects and low therapeutic efficacy [31-33]. As a viable alternative and because of their potentially supreme selectivity, a few human recombinant inhibitory antibodies are emerging as both research tools and promising therapeutic agents [34-36]. Among the currently developed anti-MT1-MMP antibodies [17, 34, 37-41], the human recombinant monoclonal DX2400 IgG is the most potent and selective inhibitory antibody raised against human MT1-MMP (Ki = 0.6 nM) [36].

We hypothesized that the antibodies that efficiently inhibit MT1-MMP should resemble TIMP-2 (the natural, most potent MT1-MMP inhibitor). TIMP-2 exhibits a long, convex-shaped loop that inserts into the protease active site and blocks the catalytic function [42, 43]. Accordingly, we suggested that the paratope/complementarity determining regions (CDRs) of a MT1-MMP-inhibitory antibody should be flexible and long enough to access the active site cavity. We then custom-designed synthetic human Fab libraries carrying a 23-27 residue long and flexible heavy chain (V_H_) CDR-H3 paratope that was inserted into the human antibody framework. Here, we characterize a novel, selective and potent, human recombinant 3A2 MT1-MMP antibody identified in our hybrid Fab antibody library [43]. The unique methodology we used in designing and selecting this inhibitory antibody is described in our accompanying manuscript (submitted). Our results support and extent the investigations by others. Our current observations demonstrate the importance of MT1-MMP in promoting the metastatic process. Conversely, the selective anti-MT1-MMP monotherapy is likely to alleviate the melanoma metastatic burden and, ultimately, to perform similarly in certain other metastatic cancers with the enhanced expression and activity of MT1-MMP.

## RESULTS

### The 3A2 Fab is an efficient inhibitor of MT1-MMP

**Figure 1:**
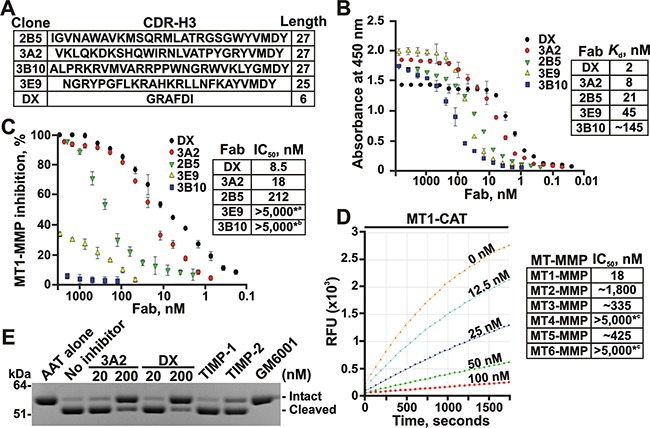
The 3A2 Fab is a selective, low nanomolar inhibitor of MT1-MMP **A.** The clone, the sequence and the length of the CDR-H3 region in the selected Fab binders of MT1-MMP. **B.** Fab ELISA with the selected Fab binders of MT1-MMP. *Left*, the biotin-labeled catalytic domain of MT1-MMP (MT1-CAT) was captured onto streptavidin-coated wells of a 96-well plate. The Fab antibodies (0-8,000 nM) were allowed to bind to MT1-CAT. The bound antibodies were detected using HRP-conjugated anti-human Fab and a TMB/E substrate. Data are means ± SE from three individual experiments performed in triplicate. *Right*, the *K*_d_ values were calculated from the reactions in which a half of MT1-CAT was complexed with the added Fab. **C.** Inhibition of the MT1-MMP cleavage activity by the selected Fab antibodies. *Left*, the dose-response inhibition by the Fab fragments. The cleavage activity of MT1-CAT (5 nM) was measured in the presence of the increasing concentrations of the Fab antibodies (0-5,000 nM) using a Mca-PLGL-Dpa-AR-NH_2_ substrate (1 μM). The residual cleavage activity was expressed in percent relative to a “no Fab” control. Data are means ± SE from 3 individual experiments conducted in triplicate. *Right*, the IC_50_ values for the selected Fab antibodies. *^a^ and *^b^, the weak inhibitory and non-inhibitory Fabs, respectively. **D.** The 3A2 Fab antibody is a selective inhibitor of MT1-MMP. The individual CAT of MT-MMPs (5 nM, each) were co-incubated with the increasing concentrations of the 3A2 Fab antibody (0-5,000 nM). The residual cleavage activity was measured using a Mca-PLGL-Dpa-AR-NH_2_ substrate (1 μM). *Left*, the representative dose-response curves of the 3A2 Fab antibody against MT1-CAT. *Right*, the IC_50_ values of the 3A2 Fab antibody in the individual MT-MMPs. *RFU*, relative fluorescence unit; *^c^, no inhibition at the highest Fab concentration used. **E.** The 3A2 Fab antibody inhibits MT1-MMP proteolysis of AAT. AAT (2 μM) was co-incubated with MT1-CAT alone (40 nM, no inhibitor) or jointly with the 3A2 or DX2400 Fab fragments (20 and 200 nM, each), TIMP-1 (1,000 nM), TIMP-2 (20 nM) or GM6001 (1,000 nM). The reactions were analyzed by SDS-PAGE followed by Coomassie staining. DX, DX2400.

We synthesized the human Fab antibody library (over 1.25×10^9^ individual variants) that exhibited the extended, 23-27 residue long, V_H_ CDR-H3 segments (submitted). These Fab constructs were expressed in *E. coli*, purified from the *E. coli* cell lysates and the purified samples (purity >95%) were then used in our studies. We next identified over twenty binders from which fourteen performed as potent inhibitors of MT1-MMP. In our current study, four of the most efficient Fab antibody binders of MT1-MMP were then selected for the in-depth analysis and characterization (Figure 1A).

Using the Fab ELISA tests with the individual catalytic domain of MT1-MMP (MT1-CAT) as bait for the increasing concentrations of the Fab fragments, we confirmed that the 3A2 (V_H_ CDR-H3 sequence VKLQKDKSHQWIRNLVATPYGRYVMDY), 2B5 (V_H_ CDR-H3 sequence IGVNAWAVKMSQRMLATRGSGWY VMDY) and 3E9 (V_H_ CDR-H3 sequence NGRY PGFLKRAHKRLLNFKAYVMDY) Fab fragments efficiently bound to MT1-MMP, while the 3B10 Fab (V_H_ CDR-H3 sequence ALPRKRVMVARRP PWNGRWVKLYGMDY) was far less efficient in our ELISA binding tests. The *K*_d_ value of the 3A2 Fab (8 nM) was comparable with that of the DX2400 Fab (2 nM; V_H_ CDR-H3 sequence GRAFDI), which is currently the most potent inhibitory antibody developed against human MT1-MMP [35, 36] (Figure 1B). Our additional cleavage tests using the Mca-PLGL-Dpa-AR-NH_2_ peptide as a cleavage substrate and the increasing concentrations of the Fab fragments as inhibitors revealed that both the 2B5 and 3A2 Fab antibodies performed as efficient, low nanomolar range, antagonists of MT1-MMP. Thus, the IC_50_ value of the 3A2 Fab was 18 nM, suggesting that this Fab sequence is only 2-fold less efficient against MT1-MMP compared with the DX2400 Fab (8.5 nM). In turn, neither the 3B10 nor 3E9 Fab fragments inhibited MT1-MMP activity (IC_50_ > 5,000 nM for both) indicating that the binding efficacy does not always directly correlate with the inhibitory potency (Figure 1C).

### The 3A2 Fab performs as a selective inhibitor of MT1-MMP

To test if the 3A2 antibody performs not only as an efficient but also as a selective inhibitor, we evaluated its off-target interactions against a panel of the purified MMPs. Because earlier we have already proved that the 3A2 antibody did not cross-react with the soluble MMP-2 and MMP-9 (submitted), here we evaluated the more closely related enzymes from the MT-MMP sub-family, including MT2-MMP, MT3-MMP, MT4-MMP, MT5-MMP and MT6-MMP. For these purposes, the increasing concentrations of the 3A2 Fab were used to inhibit the cleavage activity of these five MT-MMPs against the Mca-PLGL-Dpa-AR-NH2 substrate. Our results clearly indicated that the 3A2 Fab was highly specific against MT1-MMP (IC_50_ = 18 nM). The 3A2 Fab was incapable of inhibiting MT4-MMP and MT6-MMP (IC_50_ > 5,000 nM for both), the proteases that are less related to MT1-MMP. An insignificant inhibitory efficacy of the 3A2 Fab was observed against MT2-MMP (IC_50_ ≈ 1,800 nM), MT3-MMP (IC_50_ ≈ 335 nM) and MT5-MMP (IC_50_ ≈ 425 nM) (Figure 1D).

### Inhibition of MT1-MMP proteolysis of α1-antitrypsin serpin by the 3A2 Fab

Because the 3A2 Fab outperformed other Fab constructs, our further studies were focused on the 3A2 antibody alone. The 3A2 Fab was efficient not only in suppressing the peptide cleavage by MT1-MMP but also MT1-MMP proteolysis of the protein substrates. Thus, human α_1_-antitrypsin (AAT) serpin is a clinically relevant protein target of MMP proteolysis as well as a common and convenient substrate for testing the functional activity of MMPs *in vitro*. MMPs normally cleave the 56 kDa AAT near the C-terminus to generate the 52 kDa N-terminal and the 4 kDa C-terminal fragments [44-48]. In agreement, at a 1:50 enzyme-substrate molar ratio, MT1-CAT almost fully proteolyzed AAT in 1 h. Inhibition of MT1-MMP proteolysis was already observable at the low, 20 nM, concentration of the 3A2 antibody. A 200 nM 3A2 Fab concentration caused an almost quantitative inhibition of MT1-MMP proteolysis of AAT. Similar results were also observed with the DX2400 Fab (Figure 1E). As controls, we co-incubated AAT with TIMP-1 (an inefficient MT1-MMP inhibitor), TIMP-2 (a potent MT1-MMP inhibitor) or the broad spectrum hydroxamate MMP inhibitor GM6001 (IC_50_ = 0.4 nM against MT1-MMP). As expected, both TIMP-2 (20 nM) and GM6001 (100 nM) readily abolished MT1-MMP proteolysis of ATT, whereas TIMP-1 (1,000 nM) was without effect. In sum, under our experimental conditions the 3A2 Fab inhibitory potency was similar with that of both TIMP-2 and DX2400 Fab.

### The 3A2 Fab inhibits the functional activity of cellular MT1-MMP

**Figure 2:**
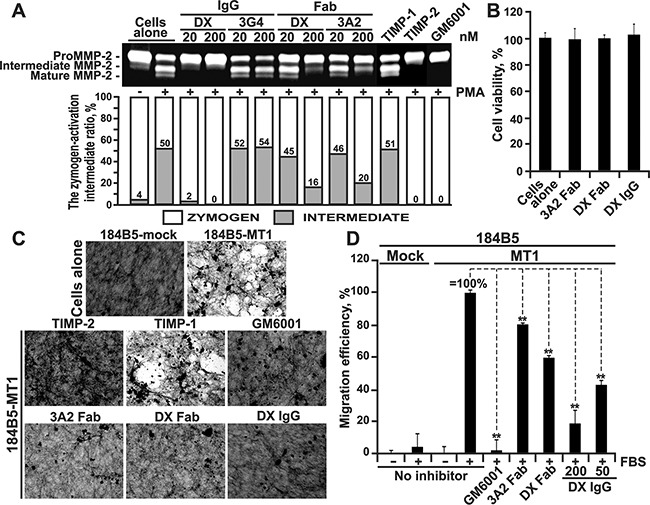
The 3A2 Fab antibody inhibits the functional activity of cellular MT1-MMP **A.** The 3A2 Fab and the DX2400 Fab and IgG antibodies inhibited activation of the proMMP-2 zymogen by cellular MT1-MMP in HT1080 cells. *Top*, to induce proMMP-2 activation, cells were stimulated using phorbol 12-myristate 13-acetate (PMA; 50 ng/ml). Cells were then co-incubated with the 3A2 and DX2400 antibodies (20-200 nM, each) and also with the non-inhibitory MT1-MMP 3G4 IgG antibody (20-200 nM), TIMP-1 (1,000 nM), TIMP-2 (100 nM) and GM6001 (1,000 nM) controls. Medium aliquots were next analyzed by gelatin zymography to identify the status of MMP-2. Cells alone, no inhibitors were added to the cells. *Bottom*, the digitized zymogen:activation intermediate ratio in the MMP-2 samples. White and grey rectangles, zymogen and activation intermediate, respectively. The numbers indicate the percentage of the activation intermediate relative to the total combined amount of the zymogen and the intermediate. **B.** The 3A2 Fab and the DX2400 Fab and IgG do not affect cell viability. Normal mammary epithelial 184B5 cells were incubated alone (cells alone) or co-incubated with the antibodies (1,000 nM, each). Cell viability was measured using a luminescent ATP-Lite assay. Data are means ± SE from three individual experiments performed in triplicate. **C.** The 3A2 Fab antibody inhibits COL-I degradation by cellular MT1-MMP. MT1-MMP-deficient 184B5-mock and MT1-MMP-overexpressing 184B5-MT1 cells were plated onto COL-I layers and then incubated alone or co-incubated for 5 days with the 3A2 Fab (200 nM), DX2400 Fab or IgG (200 nM and 100 nM, respectively), TIMP-1 (1,000 nM), TIMP-2 (100 nM) or GM6001 (1,000 nM). After the removal of cells, COL-I was stained with Coomassie. The representative images from three independent experiments performed in triplicate are shown. **D.** Cell invasion through COL-I. 184B5-mock (mock) and 184B5-MT1 (MT1) cells (1×10^5^, each) were allowed to migrate alone (no inhibitor) or in the presence of the 3A2 or DX2400 Fab fragments (500 nM, each) or the indicated concentrations DX2400 IgG. GM6001 (1,000 nM) and 10% FBS were used as a control and a chemoattractant, respectively. Migration efficiency was calculated relative to MT1 cells, no inhibitor and 10% FBS (=100%). Data are means ± SE from three individual experiments conducted in triplicate. **, P < 0.05. DX, DX2400.

Cellular MT1-MMP is a single physiological activator of the soluble MMP-2 proenzyme [49]. In the proMMP-2 activation pathway, MT1-MMP cleaves the Asn37-Leu38 scissile bond in the proMMP-2 prodomain sequence. This cleavage transforms the MMP-2 zymogen into the 64 kDa activation intermediate. The latter then autolytically generates the 62 kDa mature enzyme [49, 50]. To elucidate if the 3A2 antibody inhibits the functional activity of cellular MT1-MMP, we co-incubated human fibrosarcoma HT1080 cells that express naturally MT1-MMP with the 3A2 Fab for 16-18 h. For comparison, HT1080 cells were also co-incubated with TIMP-1, TIMP-2 or either the DX2400 Fab or IgG. The non-inhibitory MT1-MMP 3G4 antibody was used as an irrelevant antibody control. GM6001 hydroxamate was used as an additional control. To increase the MT1-MMP cellular activity, HT1080 cells were stimulated with phorbol 12-myristate 13-acetate. Conditioned medium aliquots were then analyzed by gelatin zymography. As expected, primarily the 68 kDa proMMP-2 zymogen was revealed in the intact HT1080 cells, while the significant levels of the 64 kDa activation intermediate and the 62 kDa mature enzyme of MMP-2 were present in the stimulated HT1080 cells (Figure 2A). Both GM6001 (1,000 nM) and TIMP-2 (100 nM) blocked the proMMP-2 activation in the stimulated HT1080 cells, while TIMP-1 (a poor inhibitor of MT1-MMP) and a non-inhibitory MT1-MMP 3G4 antibody were both ineffective (1,000 nM, each). Our quantification of the zymogen:activation intermediate ratio in the MMP-2 samples demonstrated a comparable inhibitory efficacy of the 3A2 and DX2400 Fab fragments (Figure 2A).

To corroborate these data and demonstrate that the inhibition of MMP-2 activation was caused by the inactivation of cellular MT1-MMP rather than by the antibody cytotoxicity, we determined if the MT1-MMP antibodies affected cell viability. For these purposes, we used normal mammary epithelial 184B5 cells which are more fragile compared with the apoptosis-resistant cancer cells. Cells were incubated for 24 h with a high, 1 μM, concentration of the antibodies. Viable cells were then assessed using an ATP-Lite assay. Our data clearly demonstrated that none of the inhibitory antibodies had an effect on cell viability (Figure 2B).

### 3A2 Fab inhibits collagenolysis mediated by cellular MT1-MMP

Because MT1-MMP is a collagenase, we next tested if the 3A2 Fab was capable of inhibiting the collagenolytic activity of cellular MT1-MMP. For these purposes, we performed a type-I collagen (COL-I) degradation assay using 184B5-MT1 cells and assessed the 3A2 inhibitory potency in comparison with that of GM6001, TIMP-1, TIMP-2, and both DX2400 Fab and IgG. As an additional control, we used 184B5-mock cells, which do not express MT1-MMP and, as a result, do not proteolyze COL-I. Cells were plated for 5 days onto slides coated with COL-I, then fixed, stained with Coomassie and observed using a microscope. 184B5-MT1 cells readily degraded COL-I, while 184B5-mock cells were negative. Both GM6001 (1,000 nM) and TIMP-2 (100 nM) blocked COL-I degradation in 184B5-MT1 cells, while TIMP-1 (1,000 nM) was inactive suggesting that MT1-MMP rather than other MMPs was a key in COL-I cleavage in our cell system. Consistently, the 3A2 Fab (200 nM) and both the DX2400 Fab (200 nM) and IgG (100 nM) quantitatively inhibited MT1-MMP-dependent collagenolysis in 184B5-MT1 cells (Figure 2C).

### 3A2 Fab inhibits MT1-MMP-dependent cell invasion

Because cellular MT1-MMP plays a major role in promoting cell invasion, we evaluated if the 3A2 antibody was capable of affecting cell invasion through a layer of COL-I. For these purposes, we performed a COL-I invasion assay using Boyden migration chambers in which porous membranes were coated with a thin layer of COL-I. In the assay we used 184B5-MT1 cells and evaluated the 3A2 Fab inhibitory potency in comparison with that of both the DX2400 Fab and IgG antibodies. The cells were plated in serum-free DMEM in the upper chamber. DMEM-10% FBS was added to the lower chamber as a chemoattractant. The inhibitors were added to both chambers. The 184B5-mock cells that do not produce MT1-MMP and, as a result, do not migrate efficiently serve as a control [51, 52]. In turn, 184B5-MT1 cells stably transfected with MT1-MMP acquired an ability to migrate through COL-I (Figure 2D). GM6001 (1,000 nM) blocked migration of 184B5-MT1 cells to a level observed in MT1-MMP-deficient 184B5-mock cells. There was a noticeable repression of migration efficiency of 184B5-MT1 cells in the presence of the 3A2 and DX2400 Fab fragments (500 nM each). The full-length DX2400 IgG, however, exhibited in a dose-dependent manner a more efficient inhibition suggesting that the Fab fragments underperform in comparison with the respective full-length antibody format.

### 3A2 Fab inhibits cellular murine MT1-MMP

**Figure 3:**
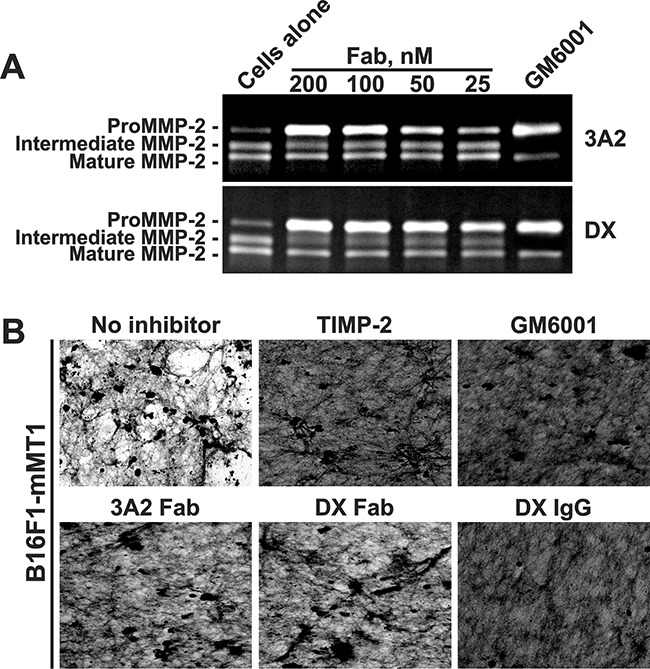
The 3A2 Fab antibody inhibits the functional activity of murine MT1-MMP **A.** Murine melanoma B16F1-mMT1 cells stably transfected with murine MT1-MMP were co-incubated with the purified proMMP-2 zymogen alone (cells alone; 5-10 nM) or jointly with the 3A2 or DX2400 Fab antibodies (25-200 nM each; *top* and *bottom* panels, respectively). Where indicated, GM6001 (1,000 nM) was added to the cells. Medium aliquots were next analyzed by gelatin zymography to identify the status of MMP-2. **B.** The 3A2 Fab antibody inhibits COL-I degradation by murine cellular MT1-MMP. B16F1-mMT1 cells were plated onto COL-I layers and then incubated alone (no inhibitor) or co-incubated for 5 days with the 3A2 Fab (200 nM), DX2400 Fab and IgG (200 nM and 100 nM, respectively), and GM6001 (1,000 nM). After the removal of cells, COL-I was stained with Coomassie. The representative images from three independent experiments performed in triplicate are shown. DX, DX2400.

Because our animal studies involve mice and because there is a four residue difference in the MT1-CAT peptide sequence in mice versus humans (Supplementary Figure S1), we determined if the anti-human 3A2 Fab was species-specific. For these purposes, we performed the MMP-2 activation assay using murine melanoma B16F1 cells with the enforced expression of murine MT1-MMP (B16F1-mMT1 cells). Because B16F1 cells do not express MMP-2 naturally, the purified proMMP-2 zymogen was added to the serum-free DMEM. Cells were then incubated in this medium with or without the 3A2 or DX2400 Fab antibodies. Medium aliquots were then analyzed by gelatin zymography. The conversion of the 68 kDa proMMP-2 into the 64 kDa activation intermediate and the 62 kDa mature enzyme was readily observed in the untreated B16F1-mMT1 cells (Figure 3A). Both the 3A2 and DX2400 Fab fragments, in a dose-dependent manner, inhibited cellular murine MT1-MMP and blocked MMP-2 activation. We also confirmed that the 3A2 and DX2400 antibodies did not affect the viability of B16F1-mMT1 cells (data not shown).

To corroborate these results, we tested if the 3A2 antibody was capable of inhibiting COL-I degradation by the cellular murine MT1-MMP. For these purposes, we plated murine B16F1-mMT1 cells onto slides coated with COL-I and then incubated the cells with or without the 3A2 Fab, the DX2400 Fab or IgG, TIMP-2 or GM6001. In 5 days, cells were removed and the slides were fixed, stained using Coomassie and observed using a microscope. Intact B16F1-mMT1 cells readily degraded COL-I layer. On a contrary, there was no significant degradation of COL-I in cells co-incubated with GM6001 (1,000 nM), TIMP-2 (100 nM) or the DX2400 antibody either in Fab or IgG format (200 nM and 100 nM, respectively) (Figure 3B). Overall, our data indicated that similar with human MT1-MMP both the 3A2 and DX2400 antibodies performed as potent inhibitors of the murine protease.

### 3A2 Fab reduces pulmonary melanoma metastasis in mice

We next evaluated the potency of the 3A2 Fab in reducing the pulmonary metastasis in the experimental melanoma metastasis model in mice. We specifically selected B16F1 cells for our *in vivo* studies because of their high metastatic propensity. To specifically focus on the MT1-MMP function in metastasis, we employed the B16F1-mMT1 cells with the enforced expression of murine MT1-MMP and the respective control B16F1-mock cells transfected with the original plasmid alone.

**Figure 4:**
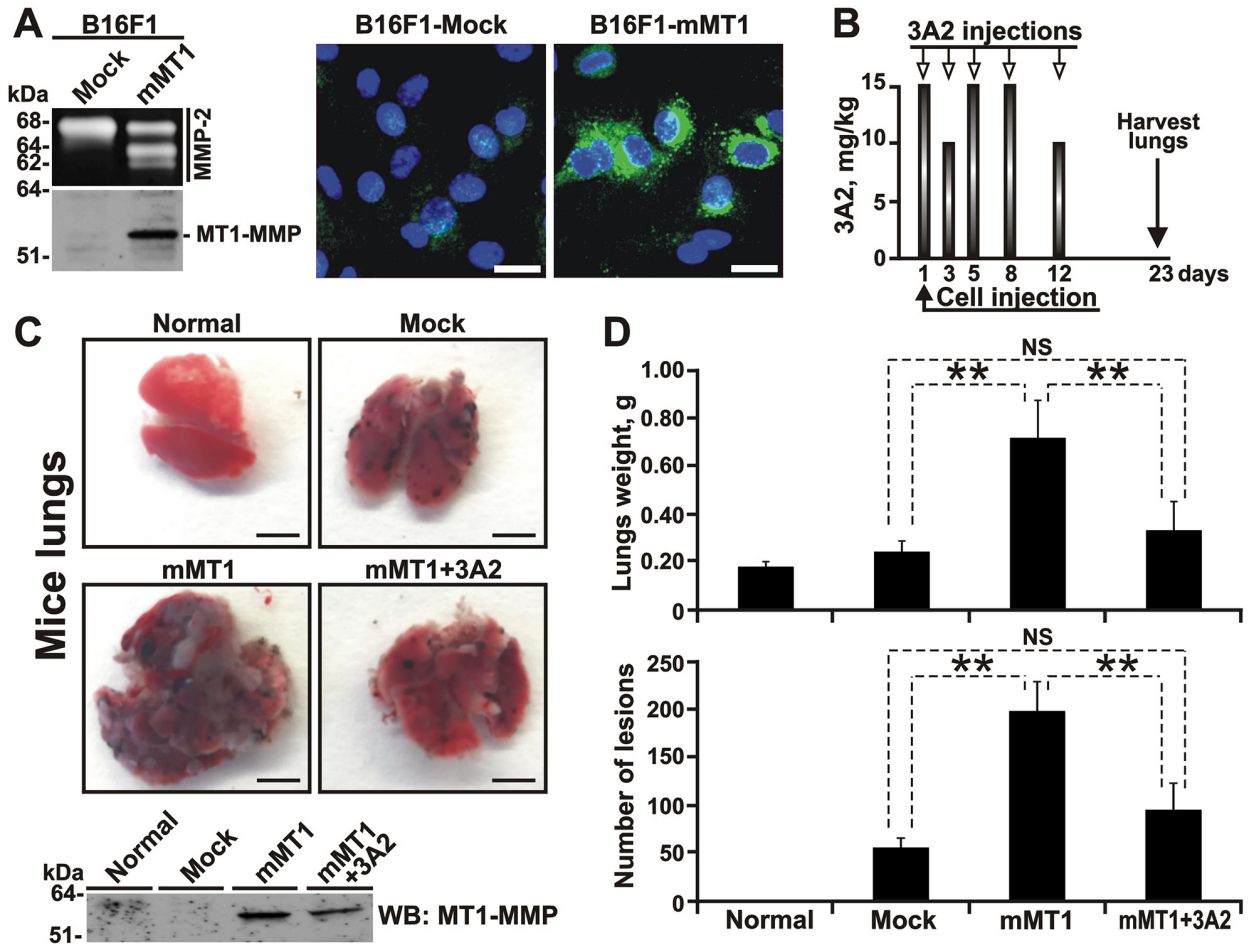
The 3A2 Fab reduces both the frequency and the size of melanoma metastatic nodules in mice **A.** The catalytically active MT1-MMP is expressed in B16F1-mMT1 cells. *Left*, the status of MMP-2 (gelatin zymography; top panel) and MT1-MMP (Western blotting with the AB8345 antibody; bottom panel) in B16F1-mock and B16F1-mMT1 cells. *Right*, the fluorescent MP-3653 reporter (25 nM) reports the presence of the catalytically active MT1-MMP (green) in B16F1-mMT1 cells but not in B16F1-mock cells. DAPI (blue). Scale bar, 10 μm. **B.** Schematic representation of our injection protocol. Athymic mice received a single tail vein injection of B16F1-mock or B16F1-mMT1 on day 1 followed by the intraperitoneal injection of the 3A2 Fab (10-15 mg/kg) on days 1-12. Mice were euthanized and the lungs harvested on day 23. **C**, *Top*, representative images of the lungs obtained from the intact control (normal), B16F1-mock (mock), B16F1-mMT1 (mMT1) and B16F1-mMT1+3A2 animal groups (mMT1+3A2). Scale bar, 5 mm. *Bottom*, Western blotting (WB) of the lung extracts (20 μg total protein each) using the MT1-MMP AB8345 antibody. **D.** The weight and the number of the pulmonary metastatic lesions in the B16F1-mock, B16F1-mMT1 and B16F1-mMT1+3A2 mice. Normal, the lungs from the intact mice control. **, P < 0.05; NS, not significant.

Multiple assays confirmed the overexpression of the functionally active MT1-MMP in B16F1-mMT1 relative to the B16F1-mock cell control. Thus, high level of MT1-MMP in B16F1-mMT1 cells was detected in cell extracts analyzed by Western Blotting with the MT1-MMP 3G4 antibody (Figure 4A). Gelatin zymography analysis of medium aliquots demonstrated that B16F1-mMT1 cells, but not the B16F1-mock control, were capable of efficiently activating MMP-2 (Figure 4A). Finally, the fluorescent MP-3653 reporter (a liposome tagged with a fluorochrome and functionalized with a PEG-5000 chain spacer linked to an inhibitory hydroxamate warhead) that binds to the active cellular MT1-MMP alone and that does not interact with the MT1-MMP proenzyme nor the catalytically inactive MT1-MMP enzyme·TIMP-2 complex [53], readily highlighted B16F1-mMT1 cells but not the control cells (Figure 4A). Based on these tests, we concluded that the control B16F1-mock cells were deficient in MT1-MMP, while the stably transfected B16F1-mMT1 cells overexpressed this membrane protease.

In our animal tests, B16F1-mMT1 cells were injected i.v. at day 1 into athymic nude mice (n=12, mMT1 mice). Mice injected with B16F1-mock cells (n=6, mock mice) served as a control. Six mice from the mMT1 group received five injections of the 3A2 Fab i.p. (10-15 mg/kg at day 1, 3, 5, 8 and 12) (Figure 4B). Six other mMT1 mice and the mock mice (n=6) received an injection i.p of vehicle alone. Additional three mice were left intact and did not receive cells nor the antibody. At day 23, mice were euthanized, and their lungs were surgically removed, weighed and photographed (Figure 4C and 4D, Supplementary Figure S2A-S2C). Western blotting analysis of the tissue extract confirmed the continuing expression of MT1-MMP in the lungs from both the mMT1 and mMT1+3A2 animal groups. In turn, the lungs of the intact and mock mice did not exhibit any noticeable levels of MT1-MMP. Because of the massive melanoma lesions, the lung weight in the mMT1 group (0.717 ± 0.160 g) greatly exceeded that in the mock animals (0.239 ± 0.047 g) and the intact mice (0.175 ± 0.023 g). In agreement, the number of metastatic nodules in the mMT1 group (198 ± 31) was approximately 4-fold higher relative to the mock control (55 ± 10). Furthermore, the nodules were bigger in size in the mMT1 mice relative to the control animals (Supplementary Figure S2A-S2B). In general, these observations agree well with the results by others [12, 13, 19] and support the pro-metastatic role of MT1-MMP in cancer. Importantly, the 3A2 antibody injections significantly reduced the lung weight (0.328 ± 0.123 g) and both the number (95 ± 28) and the size of metastatic lesions in mice from the mMT1+3A2 group when compared with the untreated mice from the mMT1 group (Figure 4D, Supplementary Figure S2B-S2C), making these parameters similar to those we recorded in the MT1-MMP-deficient mock control.

### 3A2 Fab, DX2400 Fab and TIMP-2 compete for the binding to MT1-MMP

**Figure 5:**
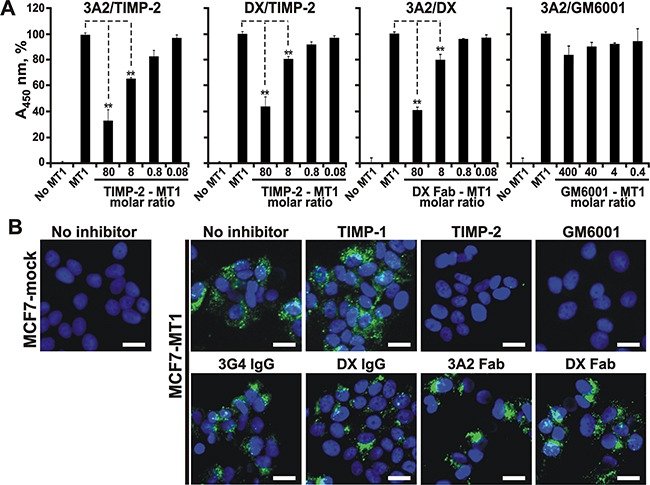
The 3A2 Fab antibody competes with TIMP-2, but not with hydroxamate inhibitor, for its binding to MT1-MMP **A.** The 3A2 and DX2400 Fab antibodies compete between themselves and also with TIMP-2 for their binding to MT1-MMP. 3A2/TIMP-2 and DX/TIMP-2, ELISA results in which the immobilized 3A2 and DX2400 Fab antibodies were each co-incubated with MT1-CAT (25 nM) and the indicated TIMP-2 – MT1-CAT molar ratios. 3A2/DX and 3A2/GM6001, ELISA results in which the immobilized 3A2 was co-incubated with MT1-CAT (25 nM) and the indicated DX2400 Fab or GM6001 – MT1-CAT molar ratio, respectively. In each ELISA, the bound MT1-MMP was then quantified using the rabbit polyclonal MT1-MMP antibody followed by the HRP-conjugated donkey anti-rabbit IgG and a TMB/E substrate. No MT1, MT1-CAT was not added. MT1, only MT1-CAT (25 nM) was added (=100%). Data are means ± SE from three individual experiments conducted in triplicate. **, P < 0.05. **B.** The 3A2 and DX2400 antibodies do not directly interact with the catalytic zinc vicinity. *Left*, the fluorescent MP-3653 reporter (25 nM) with a hydroxamate warhead did not detect the catalytically active MT1-MMP in MT1-MMP-deficient MCF7-mock cells. *Right panels*, MCF7-MT1 cells were left alone (no inhibitor) or co-incubated with the fluorescent MP-3653 reporter (25 nM) alone or jointly with the 3A2 Fab, the DX2400 Fab or IgG, the 3G4 IgG control, TIMP-1 (1,000 nM, each), TIMP-2 (50 nM) and GM6001 (100 nM). Scale bar, 10 μm. DX, DX2400.

The 3A2 Fab contained the 27-residue long, flexible V_H_ CDR-H3 to mimic the convex-shaped loop of TIMP-2 that interacts with the active site of MT1-MMP [54, 55]. To elucidate the mechanism of MT1-MMP inhibition by the 3A2 antibody and identify the 3A2 epitope, we determined if there was an overlap of the TIMP-2 binding site in the MT1-CAT molecule with that of the 3A2 and DX2400 antibodies. For these purposes, we developed several competitive ELISA methodologies. In the 3A2/TIMP-2 ELISA, the 3A2 Fab was coated on plastic and then allowed to bind to the constant amount of MT1-CAT jointly with the increasing levels of TIMP-2. The bound MT1-CAT was then measured using the rabbit MT1-MMP antibody followed by the horseradish peroxidase (HRP)-conjugated donkey anti-rabbit IgG. We observed that TIMP-2, in a dose-dependent manner, competed with the 3A2 Fab for the binding to MT1-CAT. However, even at a high, 80:1, TIMP-2 - MT1-CAT molar ratio, TIMP-2 was incapable of fully outcompeting the binding of the 3A2 Fab to MT1-CAT, thus implying that there was a partial overlap between the TIMP-2 and the 3A2 binding regions (Figure 5A). Similar observations were obtained in our DX2400/TIMP-2 ELISA that employed the immobilized DX2400 Fab (Figure 5A), suggesting an overlap among the TIMP-2 and the 3A2 and DX2400 binding regions in MT1-CAT. Our additional 3A2/DX2400 ELISA, in which the immobilized 3A2 Fab was allowed to bind to the constant amount of MT1-CAT jointly with the increasing concentrations of DX2400 Fab, confirmed that the DX2400 Fab, in a dose-dependent manner, albeit only partially, also competed the 3A2 antibody binding to MT1-CAT (Figure 5A).

### 3A2 Fab does not directly interact with the active site zinc in MT1-MMP

We next determined if the 3A2 and DX2400 inhibitory mechanism resembles that of TIMP-2 and hydroxamate inhibitors, both of which directly interact with the active site Zn^2+^ binding motif HEXXHXXGXXH in MT1-MMP [54-56]. Our 3A2/GM6001 ELISA in which the immobilized 3A2 Fab was allowed to bind to the constant concentration of MT1-CAT supplemented with the increasing concentrations of GM6001 revealed that, even at an exceedingly high, 400:1 GM6001 - MT1-CAT molar ratio, the binding of the 3A2 Fab to MT1-CAT remained unaffected (Figure 5A). This suggests that the 3A2 Fab does not interact directly with the catalytic Zn binding motif in the MT1-MMP active site.

To corroborate these results, we next determined if the 3A2 and DX2400 antibodies were able to affect the binding of the fluorescent hydroxamate-based MP-3653 reporter to cellular MT1-MMP [53]. Because of the steric hindrance between the antibody and bulky liposome-based reporter, we expected that the antibody binding would limit the concurrent binding of the reporter hydroxamate warhead to the MT1-MMP active site. In these binding experiments, we used breast carcinoma MCF7-MT1 cells stably transfected with MT1-MMP and the control MT1-MMP-deficient MCF7-mock cells. Cells were co-incubated with the MP-3653 reporter alone or jointly with the 3A2 Fab or the DX2400 in its Fab or IgG format. As controls, cells were co-incubated with the reporter in the presence of TIMP-1, TIMP-2, GM6001 or the non-inhibitory MT1-MMP 3G4 IgG antibody. The MP-3653 reporter readily bound to cell surface-associated MT1-MMP in the untreated MCF7-MT1 cells but not in MCF7-mock cells (Figure 5B). Both TIMP-2 (at a 2:1 inhibitor - reporter molar ratio) and GM6001 (at a 4:1 hydroxamate - reporter molar ratio) totally abolished the binding of the reporter to MCF7-MT1 cells, while TIMP-1 (even at a high, 40:1 inhibitor - reporter molar ratio) was inactive. In agreement, the non-inhibitory MT1-MMP 3G4 antibody also did not affect the binding of the reporter to MCF7-MT1 cells. To our surprise, neither the DX2400 Fab or IgG, nor the 3A2 Fab exhibited any significant repression of the MP-3653 reporter fluorescence in MCF7-MT1 cells. The 3A2 Fab size (≈ 75 Å in length, ≈ 50 Å in width) is >100-fold less compared with the 10 nm PEG-5000 spacer [57] of the liposome-based reporter (Supplementary Figure S3). The PEG-5000 spacer of the MP-3653 reporter is functionalized with the hydroxamate warhead which chelates the active site catalytic zinc in MT1-MMP. Accordingly, it is reasonable to expect that the hydroxamate warhead binding to the catalytic zinc did not provide any steric hindrance for TIMP-2, and, accordingly, for the 3A2 or DX2400 Fab antibodies. These results, especially if combined with our competitive ELISA tests, suggested that, in contrast with TIMP-2 and hydroxamate inhibitors, the inhibitory 3A2 and DX2400 antibodies caused MT1-MMP inactivation without any deep penetration into the active site cavity and without direct interference with the catalytic zinc ion.

### Modeling of interactions of the 3A2 Fab with MT1-MMP

The results of our binding and competition experiments, and the availability of the X-ray structures of multiple human antibodies, TIMP-2, MT1-MMP and MT1-MMP·TIMP-2 complex stimulated us to build a crude model of the 3A2 Fab - MT1-CAT interactions. To estimate the space occupied by the 3A2 Fab and TIMP-2 relative to MT1-CAT, we used as templates the structures of the MT1-MMP·TIMP-2 complex (PDB 1BQQ), of an anti-TDRD3 Fab complexed with the tudor domain of human TDRD3 (PDB 3PNW) and of GM6001 bound to the anthrax toxin lethal factor (PDB 4PKW). To model the 3A2 Fab structure, we used the residue sequences of the V_L_ and V_H_ chains of the anti-TDRD3 Fab [58] as a template. We next replaced the original anti-TDRD3 sequences Y^91^GYPI^95^ in V_L_ CDR-L3, F^29^SSSSI^34^ in V_H_ CDR-H1, S^50^ISSSYGYTY^59^ in V_H_ CDR-H2 and T^99^VRGSKKPYFSGWAMDY^115^ in V_H_ CDR-H3 with the respective V_L_ and V_H_ CDR sequences of the 3A2 Fab (SSYSLIT, LSYSSM, SIYPYSGYTY and VKLQKDKSHQWIRNLVATPYGRYVMDY, respectively) (Table 1).

**Table 1:** The modified complementary determining regions (CDR) sequences in the light (L) and the heavy (H) chains of the 3A2 Fab

CDR	Sequences of original Fab used as a template	Modified sequences in the 3A2 Fab
CDR-L3	YGYPI	SSYSLIT
CDR-H1	FSSSSI	LSYSSM
CDR-H2	SISSSYGYTY	SIYPYSGYTY
CDR-H3	TVRGSKKPYFSGWAMDY	VKLGKDKSHQWIRNLVATPYGRYVMDY

Earlier we reported that the binding of the 3A2 Fab to MT1-CAT was affected by the F260A mutation in the MT1-MMP sequence. Other mutations, including T190A, F198A, Y203A, F204A and N231A (all residues are within a 15 Å distance from the catalytic Zn^2+^ atom), did not affect the antibody binding to the protease (Supplementary Figure S1) (submitted). These data allowed us to restrict the docking area in MT1-MMP. Accordingly, we selected the N^225^EDLN^229^, S^250^SDPS^254^ and F^260^YQWMDTEN^268^ surface regions in the MT1-MMP structure as the 3A2 potential epitopes. Conversely, the SSYSLIT, LSYSSM, SIYPYSGYTY and VKLQKDKSHQWIRNLVATPYGRYVMDY V_L_ and V_H_ CDR sequences represented the potential 3A2 Fab paratopes.

**Figure 6:**
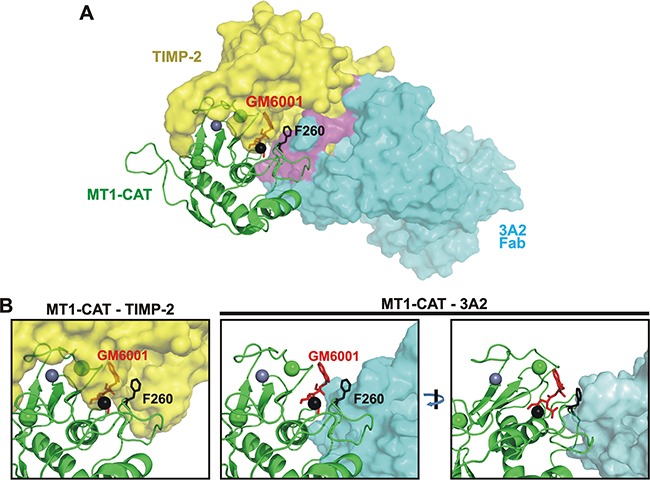
The 3A2 Fab competes with TIMP-2 binding to MT1-CAT **A.** The predicted structure of the hypothetical MT1-CAT·TIMP-2·3A2 Fab·GM6001 quadri-molecular complex. MT1-CAT is shown as cartoon (green), TIMP-2 and the 3A2 Fab are shown as yellow and cyan surfaces. GM6001, red sticks; the Phe260 residue of the MT1-CAT sequence, black sticks; the catalytic and structural zinc ions in MT1-CAT, black and grey spheres, respectively; the structural calcium ion, green sphere. A putative region where TIMP-2 clashes with the 3A2 moiety is shown in purple. The figure summarizes a detailed superimposition analysis of the available crystal structures of the tudor domain of human TDRD3 in complex with an anti-TDRD3 Fab (PDB 3PNW), MT1-CAT complexed with TIMP-2 (PDB 1BQQ) and the anthrax toxin lethal factor bound to GM6001 (PDB 4PKW). **B**, Unlike TIMP-2, the 3A2 Fab does not bind to the catalytic zinc vicinity in MT1-MMP. *Left*, close-up of the hypothetical MT1-CAT·TIMP-2·GM6001 complex shows that the bound GM6001 penetrates into the space occupied by TIMP-2 [46, 48, 49]. As a result, TIMP-2 and GM6001 compete for their binding to MT1-MMP. *Right*, two rotated close-ups of the MT1-CAT·3A2 Fab ·GM6001 complex clearly indicate that the 3A2 Fab cannot interact with the catalytic zinc vicinity (black sphere) in the MT1-MMP active site. As a result, the 3A2 Fab did not compete with GM6001 for the binding to MT1-CAT.

We then modeled a putative quadri-molecular complex that involved TIMP-2, GM6001, MT1-CAT and the designed 3A2 Fab. According to our modeling, the top scored position indicated that there was an overlap of the 3A2 Fab moiety with the space occupied by TIMP-2 in the MT1-MMP molecule (Figure 6A). These results correlated well with the partial competition between TIMP-2 and the 3A2 Fab we observed in our competitive ELISA assays (Figure 5A). Our model also indicated that TIMP-2, but not the 3A2 Fab, interacted with the catalytic Zn^2+^ in the MT1-MMP core, and, as a result, there was an expected overlap of GM6001 with the TIMP-2 structure (Figure 6B). These observations are in agreement with the results by others [29, 54-56] as well as the data from our ELISA and cell-based tests (Figure 5A, 5B). To validate these data, we are currently in the process of transforming the 3A2 Fab into its full-length IgG format. We will then determine the crystal structure of the MT1-CAT·3A2 IgG complex to better understand the molecular mechanism of MT1-MMP inhibition by the 3A2 antibody.

## DISCUSSION

Proteases, including MMPs, are both valuable diagnostic markers and pharmacological targets. Accordingly, the MMP inhibitors are expected to be valuable drugs in multiple pathologies and, especially in cancer. Ubiquitous pro-invasive MT1-MMP is an archetype membrane-associated MMP and a focus of numerous extensive studies leading to an appreciation of this protease key functions in cell migration and metastasis [59]. Naturally, this cell surface-associated protease gradually became a promising drug target. However, because of homology in the active site region of MMPs, the small-molecule active site-targeting inhibitors (primarily, hydroxamates that chelate the catalytic zinc atom in the MMP active site) cross-reacted with multiple MMPs rather than with MT1-MMP alone [29]. Off-target effects and low net efficacy of these inhibitors caused in their failure in clinical trials [30-32]. As a result, it is now broadly accepted that high level of specificity is required for pharmacological targeting of MT1-MMP.

Accordingly, high selectivity is a key parameter in a design of a successful anti-MT1-MMP therapy. Function-blocking antibodies represent a valuable alternative to small-molecule MMP inhibitors. Several MT1-MMP antibodies, both murine and human, have been recently developed and partially characterized [17, 34-36, 40, 41]. All of these antibodies target the exosites rather than the catalytic site region of MT1-MMP. Antibody targeting of the active site region that is buried in the MMP globule is a challenge, especially if the conventional approaches are employed in the library construction and antibody design.

To overcome this challenge, we designed a human Fab antibody library in which the antibody constructs exhibited the long, 23-27 residue, V_H_ CDR-H3 [43]. The length of these CDR significantly exceeded that (9-12 residues) in human and murine antibodies [60] and correlated with an average CDR size recorded in the camelid antibodies [61]. Using MT1-CAT as bait, we identified over 20 binders from which 14 performed as inhibitors of MT1-MMP rather than as broad-specificity antagonists. The most efficient and selective inhibitor was the 3A2 Fab that we extensively characterized and reported here.

Our binding and inhibitory *in vitro* and cell-based tests and assays convincingly demonstrated that the 3A2 antibody is both an efficient and selective inhibitor of cellular MT1-MMP rather than a broad-specificity MMP inhibitor. According to our tests, the selectivity and efficiency of the 3A2 Fab was similar to that of the DX2400 Fab, the most potent and selective human function-blocking anti-MT1-MMP antibody. The conversion of the DX2400 Fab into the full-length human IgG caused a further 10-fold increase in the antibody potency. Therefore, we expect that a similar improvement would take place with the 3A2 Fab fragment, suggesting that the 3A2 Fab is a very promising lead antibody against pro-tumorigenic/metastatic MT1-MMP.

Interestingly, the 3A2 antibody binding mode was dissimilar from that of hydroxamates that chelate the active site catalytic zinc in MT1-MMP and of natural protein inhibitors (such as TIMP-2) the inhibitory loop of which penetrates deeply into the protease active site pocket [54-56]. According to our binding, competition and modeling studies, the 3A2 Fab epitope only partially overlaps with the TIMP-2 binding site in the MT1-MMP catalytic domain and does not reach out to the catalytic zinc proximity. We believe that our modeling provided a structural rationale for our experimental results and sharpened a focus for our on-going mutagenesis and antibody fine-tuning efforts. Taken together, our studies generated a roadmap for the subsequent mutagenesis and structure-based affinity maturation of the 3A2 antibody.

Furthermore, because of its selectivity and low nanomolar inhibitory potency, the 3A2 antibody represents a valuable tool for the analysis of the MT1-MMP functional significance in cancer. Earlier work by others demonstrated that in a model of advanced peritoneal ovarian cancer, MT1-MMP-dependent invasion and metastasis was effectively inhibited by i.p. administration of the anti-MT1-MMP monoclonal antibody [17]. Similarly, another selective, albeit distinct, anti-MT1-MMP monoclonal antibody repressed metastasis of breast carcinoma MDA-MB-231 cells in a mouse orthotopic xenograft model [34]. We then used the 3A2 Fab to assess if the low, 10-15 mg/kg, antibody amount affected the development of metastatic lesions in the experimental pulmonary melanoma metastasis model in mice. Our results provided experimental evidence that the anti-MT1-MMP monotherapy caused a significant reduction in both the number and the size of melanoma pulmonary metastases. Our results, especially if combined with the results by others [11-13, 19], suggest that MT1-MMP plays the most critical role in the metastatic invasion rather than in tumor development and growth. Accordingly, we now believe that therapeutic targeting of MT1-MMP would be most beneficial for patients suffering from metastatic cancer and that our data would contribute to the design of the future anti-MT1-MMP clinical trials.

## MATERIALS AND METHODS

### General reagents

The reagents were purchased from Sigma-Aldrich (St. Louis, MO) unless indicated otherwise. The [(7-methoxycoumarin-4-yl)acetyl]-Pro-Leu-Gly-Leu- [N-3-(2,4-dinitrophenyl)-L-α,β-diaminopropionyl]-Ala-Arg-NH_2_ (MCA-PLGL-Dpa-AR-NH_2_) fluorogenic substrate was acquired from R&D Systems (Minneapolis, MN). Both murine monoclonal and rabbit polyclonal MT1-MMP antibodies (3G4 and AB8345, respectively), human α_1_-antitrypsin (AAT) and a broad spectrum hydroxamate inhibitor of MMPs (GM6001) were purchased from EMD Millipore (Temecula, CA). TMB/E substrate was from SurModics (Eden Prairie, MN). The mammary epithelial cell growth medium (MEGM) that included a bovine pituitary extract supplement and DMEM were from Lonza (Walkersville, MD) and Thermo Fisher Scientific (Waltham, MA), respectively. The MT1-MMP MP-3653 fluorescent reporter that exhibited an inhibitory hydroxamate warhead was described earlier [53]. Human TIMP-1 was purchased from Life Technologies (Carlsbad, CA). The DX2400 function-blocking human full-length IgG1 (DX2400 IgG) antibody was kindly provided to us by Kadmon (New York, NY).

### Cells

Human breast carcinoma MCF-7, fibrosarcoma HT1080 and mammary epithelial 184B5 cells were obtained from ATCC (Manassas, VA). Mouse melanoma B16F1 cells were a generous gift by Ralph A. Reisfeld (The Scripps Research Institute, La Jolla, CA). MCF-7, HT1080 and B16F1 cells were routinely maintained in DMEM supplemented with 10% FBS and gentamicin (10 μg/ml). Both B16F1 cells stably transfected with the original pcDNA3-zeo vector (B16F1-mock cells) or the pcDNA3-zeo plasmid encoding the full-length murine MT1-MMP proenzyme (B16F1-mMT1 cells) and both MCF-7 cells stably transfected with the empty pcDNA3-zeo vector (MCF7-mock cells) or the pcDNA3-zeo plasmid encoding the full-length human MT1-MMP (MCF7-MT1 cells) were obtained earlier [62, 63]. 184B5 cells were routinely maintained in MEGM-5% FBS supplemented with bovine pituitary extract (26 μg/ml) and gentamicin. 184B5 cells stably transfected with the original pLenti6/V5-D-TOPO lentiviral vector (184B5-mock cells) or the lentiviral vector encoding the MT1-MMP C-terminally tagged with a V5 tag (185B5-MT1 cells) were constructed earlier [51].

### Expression and purification of TIMP-2 and MMPs

The cloning, expression and purification of the recombinant human TIMP-2 was performed as previously described [53]. The TIMP-2-free proMMP-2 zymogen was isolated from the p2AHT1A72 cells (a derivative of HT1080 cells transfected with both the adenoviral E1A gene and the full-length MMP-2 cDNA) [50]. The individual catalytic domains (CAT) of human MT1-MMP, MT2-MMP, MT3-MMP, MT4-MMP, MT5-MMP and MT6-MMP were expressed in *E. coli* One Shot BL21 Star (DE3) (Thermo Fisher Scientific). The purified proteases were then isolated from the inclusion bodies using metal-chelating chromatography and refolded to restore their native conformation [45]. Only the samples with the purity >95% were used in our subsequent studies. The refolded MT-MMPs were readily used in activity assays. The concentration of the catalytically active MT-MMP samples was measured using a fluorescent assay by titration against a standard GM6001 solution of known concentration and Mca-PLGL-Dpa-AR-NH_2_ as a substrate. The steady-state rate of the substrate cleavage was plotted as a function of inhibitor concentration and fitted with the equation V=SA(E_0_−A(E{(E_0_+I+*K_i_*)−[(E_0_+I+*K_i_*)^2^−E(_0_I]^0.5^}), where V, SA, E_0_, I and *K_i_* are the steady-state rate of substrate hydrolysis, specific activity (rate per unit of MMP concentration), MT-MMP concentration, inhibitor concentration and the dissociation constant of the MT-MMP·inhibitor complex, respectively [64, 65].

### Purified Fab fragments

The cloning, expression and isolation of the DX2400 Fab fragment was reported earlier [66]. Both the DX2400 full-length IgG and Fab samples were used in our experiments for comparison purposes. From the twenty purified human Fab constructs that were capable of binding to MT1-MMP (submitted), the 2B5, 3A2, 3B10 and 3E9 Fab constructs were further characterized in this study.

### Protease inhibition assay

The cleavage assay was performed in triplicate in wells of a 96-well plate using the purified individual CAT of MMPs (5 nM) and the fluorescent peptide Mca-PLGL-Dpa-AR-NH_2_ substrate (1 μM) in 0.2 ml 50 mM HEPES, pH 7.5, containing 10 mM CaCl_2_, 0.5 mM MgCl_2_ and 10 μM ZnCl_2_. Prior to the reactions, increasing concentrations of the Fab antibodies (0-5,000 nM) were co-incubated with the protease samples for 30 min at ambient temperature. Initial reaction velocity was monitored continuously at *λ*_ex_=320 nm and *λ*_em_=400 nm using a fluorescence spectrophotometer. The IC_50_ values were calculated by determining the inhibitor concentrations that inhibited the cleavage activity by 50%. SigmaPlot was used as fitting software.

### Cleavage of AAT

The cleavage reactions (20 μl each, 1 h at 37°C) contained AAT (2 μg, ≈2 μM) and MT1-CAT (40 nM, 1:50 enzyme-substrate molar ratio) in 50 mM HEPES, pH 7.5, supplemented with 10 mM CaCl_2_ and 50 μM ZnCl_2_. Where indicated, the Fab antibodies (20-200 nM), TIMP-1 (1,000 nM), TIMP-2 (20 nM) or GM6001 (1,000 nM) were added to the reactions. The reactions were stopped using 5% SDS and analyzed by SDS-PAGE in a 4-12% gradient NuPAGE-MOPS gel (Life Technologies) followed by Coomassie Blue R250 staining.

### Cell viability assays

Assays were conducted in wells of a 96-well flat bottom, white wall plates. 184B5 and B16F1-mMT cells (7.5×10^4^) were grown for 16 h in MEGM-5% FBS and DMEM-10% FBS, respectively. After washing with PBS, fresh FBS-free medium (0.1 ml) containing the Fab antibodies (1,000 nM) was added to the cells and incubation was continued for an additional 24 h. The viable cells were then counted using a luminescent ATP-Lite assay (PerkinElmer; Waltham, MA). Each datum point represents the results of at least 2 independent experiments performed in triplicate.

### MMP-2 gelatin zymography and Western blotting

Following incubation of the cells (1×10^5^/well of a 48-well plate) in serum-free medium (150 μl), the status of MMP-2 was analyzed by gelatin zymography of the medium aliquots (15 μl) using precast 10% acrylamide gels co-polymerized with 0.1% gelatin (Life Technologies) as described previously [53]. To stimulate the MMP-2 activation, HT1080 cells (1×10^5^) were stimulated for 24 h using phorbol 12-myristate 13-acetate (50 ng/ml) with or without the presence of the Fab antibodies (20-200 nM), TIMP-1 (1,000 nM), TIMP-2 (100 nM) or GM6001 (1,000 nM). We also used the B16F1-mMT1 cells that expressed the murine MT1-MMP and the respective control B16F1-mock cells transfected with the original plasmid alone. In the latter, cells (1×10^5^) were seeded for 24 h in DMEM-10% FBS in wells of a 48-well plate. Cells were replenished with fresh DMEM (150 μl) containing purified proMMP-2 (5-10 nM) alone or jointly with the Fab antibodies (25-200 nM) or GM6001 (1,000 nM). In 18 h, the medium aliquots (15 μl) were analyzed by gelatin zymography, while cells were washed with PBS and then lysed in TBS containing 50 mM N-octyl-β-D-glucopyranoside, 1 mM phenylmethylsulphonyl fluoride, 10 mM EDTA, and a protease inhibitor cocktail set III. Insoluble material was removed by centrifugation (14,000×g; 30 min). The supernatant aliquots (5 μg total proteins) were separated by electrophoresis in a 4–12% gradient NuPAGE-MOPS gel (Life Technologies) and analyzed by Western blotting with the MT1-MMP AB8345 antibody followed by the secondary HRP-conjugated antibody (Jackson ImmunoResearch; West Grove, PA) and a SuperSignal West Dura Extended Duration Substrate kit (Thermo Fisher Scientific). Where indicated, the images were digitized and the intensity of the bands was quantified using ImageJ software. These data were used to measure the zymogen:activation intermediate ratio of MMP-2 expressed as a percentage of the zymogen and the activation intermediate each related to their combined total amount.

### COL-I degradation assay

The assay was performed in triplicate in wells of a 24-well plate. Wells were coated for 4 h at 37°C with neutralized, chilled rat tail COL-I (300 μg/ml, 350 μl in PBS) and then air dried for 16 h. The COL-I coating was washed twice for 30 min at ambient temperature with sterile H_2_O and rehydrated for 2 h at 37°C in 0.4 ml DMEM. Seeded cells (1×10^5^) were allowed to attach for 4 h. Fresh DMEM (0.4 ml) containing the 3A2 Fab (200 nM), the DX2400 Fab or IgG antibodies (100-200 nM), TIMP-1 (1,000 nM), TIMP-2 (100 nM) or GM6001 (1,000 nM) was then added to the cells. At day 3, cells were replenished with fresh medium supplemented with the respective inhibitors and incubation was continued for an additional 2 days. Cells were next detached with 0.25% trypsin/0.5 mM EDTA. COL-I was fixed using 4% *p*-formaldehyde and stained with Coomassie Blue R250. The images were captured using a Nikon TE-2000 microscope with a ×20 objective and a CCD camera. COL-I degradation appeared as clear zones in the blue background.

### Cell invasion assays

The assays were conducted in wells of a 24-well, 8 μm pore size Transwell plate (Corning; Corning, NY). A 6.5-mm insert membrane was coated using 0.1 ml rat tail COL-I (0.3 mg/ml; BD Biosciences; Franklin Lakes, NJ), air dried for 16-18 h and then rehydrated for 1 h in 0.2 ml DMEM. The inner/lower chamber contained DMEM-10% FBS as a chemoattractant. The cells (1×10^5^) were co-incubated for 60 min in DMEM alone or supplemented with the 3A2 and DX2400 Fab fragments (500 nM, each), DX2400 IgG (50-200 nM) or GM6001 (1,000 nM) prior to plating cells into the outer/upper chamber. The inhibitor concentration was identical in both the outer and inner chambers. The cells were allowed to migrate for 16-18 h. The cells were then removed from the membrane top surface using a cotton swab. The cells on the membrane bottom surface were fixed and stained using 0.2% crystal violet-20% methanol. The incorporated dye was extracted using 1% SDS and the A_590_ of the extract was measured using a microplate reader. Data are means ± SE from 3 individual experiments conducted in triplicate. Cell invasion level was calculated relative to the intact 184B5-MT1 cells (=100%).

### Biotinylation of MT1-CAT

The refolded MT1-CAT aliquot (0.2 mg/ml in 0.7 ml 50 mM HEPES pH 7.5) was labeled for 30 min on ice at a 1:20 enzyme-biotin molar ratio using EZ-Link sulfo-NHS-LC-biotin (Thermo Fisher Scientific). Excess biotin was removed using a 0.7-ml protein desalting spin-column.

### Fab antibody binding to MT1-CAT measured by ELISA

The wells of a 96-well Maxisorp ELISA plate (Nunc; Rochester, NY) were coated with Streptavidin (3 μg/ml, 125 μl 15 mM bicarbonate buffer, pH 9.6) at 4°C for 18 h, then blocked with 0.5% gelatin in TBS-0.075% Tween (TBS/T) for 1 h at 37°C. Following two washes with TBS/T, the plate was incubated for 20 min at ambient temperature with the biotinylated MT1-CAT sample (25 nM). The unbound MT1-CAT was removed using multiple washes with TBS/T (5 min each). Increasing concentrations of the Fab antibodies (0-8,000 nM; 50 μl TBS/T-0.5% gelatin) were allowed to bind to MT1-CAT for 1 h at ambient temperature. Following extensive washing with TBS/T, HRP-conjugated goat anti-human Fab (dilution 1:10,000; 50 μl TBS/T-0.5% gelatin) was added to the wells and incubation continued for an additional 1 h. Following extensive washing with TBS/T and then with H_2_O, TMB/E substrate (0.1 ml) was added to the wells. The reaction was stopped using 1 M H_2_SO_4_ (25 μl). The resulting A_450_ values were measured using a plate reader. The *K*_d_ values were calculated by determining the inhibitor concentrations that bound 50% of the MT1-MMP molecules. SigmaPlot was used as fitting software. Statistical analyses were performed using a two-tailed, unpaired Student's t-test. P values below 0.05 were considered significant. Data are means ± SE from at least 3 individual experiments performed in triplicate.

### Competition between the Fab antibodies and TIMP-2 for the binding to MT1-CAT measured by ELISA

Wells of a 96-well Maxisorp ELISA plate were coated with the 3A2 or DX2400 Fab aliquots (2 μg/ml each, 125 μl 15 mM bicarbonate buffer, pH 9.6) at 4°C for 18 h and then blocked with 3% BSA in PBS-0.075% Tween (PBS/T) for 1 h at 37°C. The follow-on procedures were carried out at ambient temperature. Following washes with PBS/T, MT1-CAT (25 nM; PBS/T-1% BSA) alone or jointly with increasing concentrations of TIMP-2 (2-2,000 nM), DX2400 Fab (2-2,000 nM) or GM6001 (10-10,000 nM) was added to the wells and incubation continued for 2 h. The unbound material was removed using multiple washings in PBS/T (5 min each) and then the rabbit MT1-MMP Ab8345 antibody (0.5 μg/ml; 0.1 ml PBS/T-1% BSA) was added for 2 h. Following extensive washing with PBS/T, the HRP-conjugated donkey anti-rabbit IgG (1:10,000 dilution, 0.1 ml PBS/T-1% BSA) was added to the wells and incubation continued for an additional 1 h. After extensive washing with PBS/T and then with H_2_O, TMB/E substrate (0.1 ml) was added to the wells. The reaction was stopped by adding 1 M H_2_SO_4_ (0.1 ml) and the resulting A_450_ value was measured using a plate reader. Data are means ± SE from at least 3 individual experiments performed in triplicate.

### Cell-based assay using the fluorescent MP-3653 reporter

Cells were plated in DMEM-10% FBS on a 15-mm glass coverslip and allowed to reach a 25-50% confluency. The cells were then washed in PBS and co-incubated for 30 min at 37°C in DMEM supplemented with either 0.2% BSA alone or jointly with the 3A2 Fab, the DX2400 Fab or IgG format, the 3G4 IgG control, TIMP-1 (1,000 nM, each), TIMP-2 (50 nM) or GM6001 (100 nM). The MP-3653 reporter (25 nM) was next added to the cells and incubation continued for an additional 3 h. Cells were then washed in PBS and fixed in 4% *p*-formaldehyde, mounted in the VectaShield mounting medium containing DAPI for the nuclear staining (Vector Lab; Burlingame, CA) and examined using a fluorescence microscope equipped with a digital camera.

### Melanoma pulmonary metastasis in mice

To readily develop pulmonary metastatic lesions, at day 1, 5-6 week-old female athymic Foxn1nu *nude* mice (Envigo; Indianapolis, IN) received a single tail vein injection of B16F1-mock and B16F1-mMT1 cells [0.2×10^6^ in 0.2 ml Hank's Balanced Salt Solution (HBSS)] [62, 67]. Six and twelve animals received B16F1-mock and B16F1-mMT1 cells, respectively. Additional 3 mice were left intact and served as a control for normal behavior and the normal lung weight. At day 1, 3, 5, 8 and 12, six animals from the B16F1-mMT1 group also received an intraperitoneal injection (i.p.) of the 3A2 Fab (10-15 mg/kg in 150 μl HBSS), while other mice received the vehicle alone. At day 23, mice were euthanized according to the NIH guidelines. The lungs were harvested, washed in ice-cold PBS and weighed. For each mouse, the lungs were photographed and then sectioned (Supplementary Figure S2A-S2C). Metastatic nodules were counted using the digitized lobe images. The lung samples were next snap-frozen. The sections (0.15 mg each) of the lungs were extracted in 0.9 ml 20 mM Tris-HCl, pH 7.4, supplemented with 150 mM NaCl, 0.5% deoxycholate, 1% IGEPAL, 1% Triton X-100, 0.1% SDS, a protease inhibitor cocktail set III, 1 mM phenylmethylsulfonyl fluoride, 10 mM EDTA and 10 μM GM6001. The solubilized material was separated from the pellet by centrifugation (14,000×g; 30 min). The protein concentration was then adjusted in the samples to reach 3 mg/ml. Sample aliquots (20 μg total protein each) were then analyzed by Western blotting with the MT1-MMP AB8345 antibody followed by the secondary HRP-conjugated antibody and a SuperSignal West Dura Extended Duration Substrate kit. All protocols for animal studies were reviewed and approved by the Institutional Animal Care and Use Committee at SBP Medical Discovery Institute.

### Structural modeling

In our modeling studies, we used the crystal structure of an anti-TDRD3 Fab in its complex with the tudor domain of human TDRD3 (PDB 3PNW) [58], MT1-CAT complexed with TIMP-2 (PDB 1BQQ) [54] and the GM6001 hydroxamate bound to the anthrax toxin lethal factor (PDB 4PKW) [68]. The structures were analyzed and superimposed using PyMOL. The 3A2 Fab fold was engineered using the peptide sequence of the V_L_ and V_H_ chains of the anti-TDRD3 Fab [58] as a template. In the latter, the sequences V_L_ CDR-L3 (Y^91^GYPI^95^), V_H_ CDR-H1 (F^29^SSSSI^34^), V_H_ CDR-H2 (S^50^ISSSYGYTY^59^) and V_H_ CDR-H3 (T^99^VRGSKKPYFSGWAMDY^115^) were replaced by SSYSLIT, LSYSSM, SIYPYSGYTY and VKLQKDKSHQWIRNLVATPYGRYVMDY, respectively (Table 1). We then combined the structure of the V_L_ and V_H_ chains from 3PNW with the structure of the modified CDR-L3, CDR-H1, CDR-H2 and CDR-H3 regions. The structure of the modified CDR regions was modeled using the server version of the Modeller program available from ModBase [69] and the crystal structure of human prolactin receptor complexed with Fab (PDB 4I18) [70]. Using the FATCAT program [71], the modeled V_H_ chain of the 3A2 Fab was then reoriented to the position of the V_H_ chain present in the 3PNW structure. The resulting structure was then used in our docking experiments. The protein-protein docking was performed using ZDOCK on-line server [72]. For restricting docking area of MT1-MMP with the 3A2 Fab, we selected N^225^EDLN^229^, S^250^SDPS^254^ and F^260^YQWMDTEN^268^ sequences as MT1-MMP epitopes and the SSYSLIT, LSYSSM, SIYPYSGYTY and VKLQKDKSHQWIRNLVATPYGRYVMDY modified sequences in both CDRs V_L_ and V_H_ chains of the Fab as paratopes. Figure 6 shows the top scored, most probable relative fold of the hypothetical MT1-MMP·TIMP-2·3A2 Fab·GM6001 quadri-molecular complex.

## SUPPLEMENTARY MATERIALS FIGURES


