# Supplementary material for: Selective function-blocking monoclonal human antibody highlights the important role of membrane type-1 matrix metalloproteinase (MT1-MMP) in metastasis
Source: Oncotarget. 2016 Jul 11;8(2):2781–99. doi: 10.18632/oncotarget.13157 (PMC5356841; doi:10.18632/oncotarget.13157)
Supplement: Supplementary file 1 [file oncotarget-08-2781-s001.pdf]

Selective function-blocking monoclonal human antibody highlights the important role of membrane type-1 matrix metalloproteinase (MT1-MMP) in metastasis

SUPPLEMENTARY FIGURES

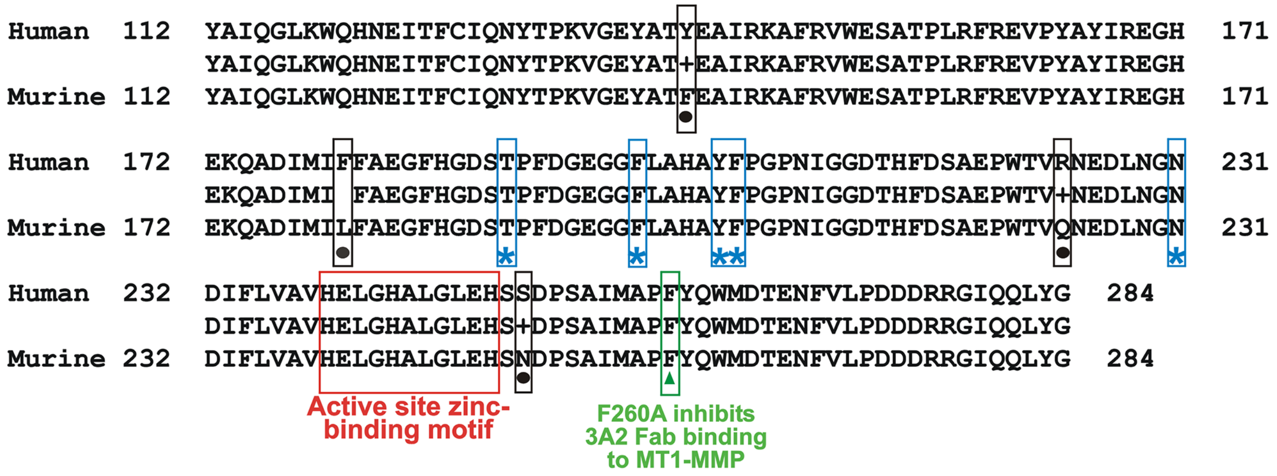

Supplementary Figure S1: Sequence alignment of the catalytic domain of human (*top*) and murine (*bottom*) MT1-MMP. Black dots denote the four species-specific residues. The HExxHxxGxxH active site zinc-binding motif, red square. The mutation F260A that affects the binding of the 3A2 Fab to MT1-MMP, green triangle (submitted). The mutations (T190A, F198A, Y203A, F204A and N231A) that do not interfere with the 3A2 binding to MT1-MMP, cyan asterisks. Note that the four species-specific residues do not affect TIMP-2 binding to MT1-MMP [47].

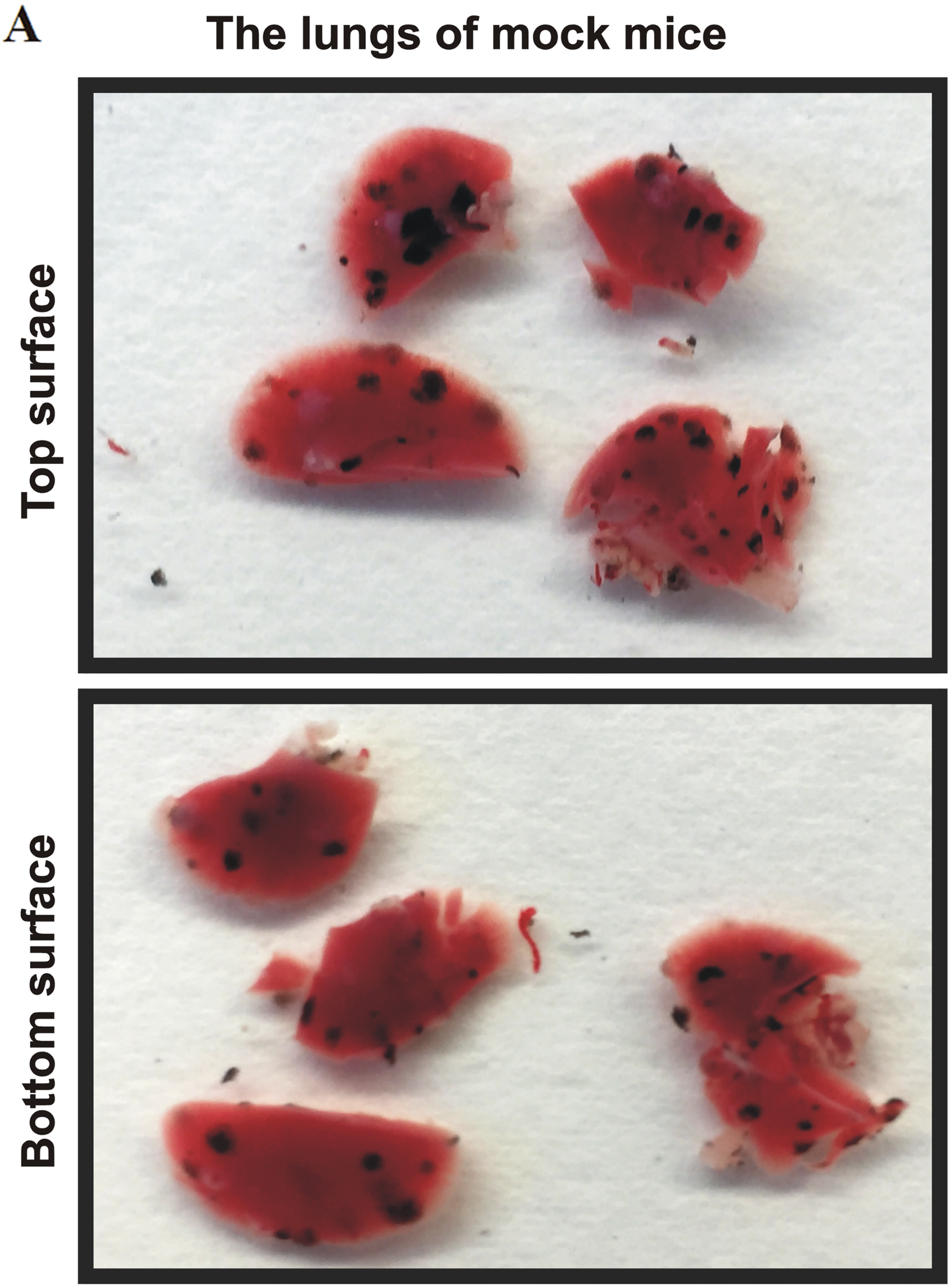

Supplementary Figure S2: Metastatic nodules in mice. A. Pulmonary melanoma metastatic nodules were counted at day 23 post-cell injection in mice from the B16F1-mock (mock) group. Representative images are shown. (*Continued*)

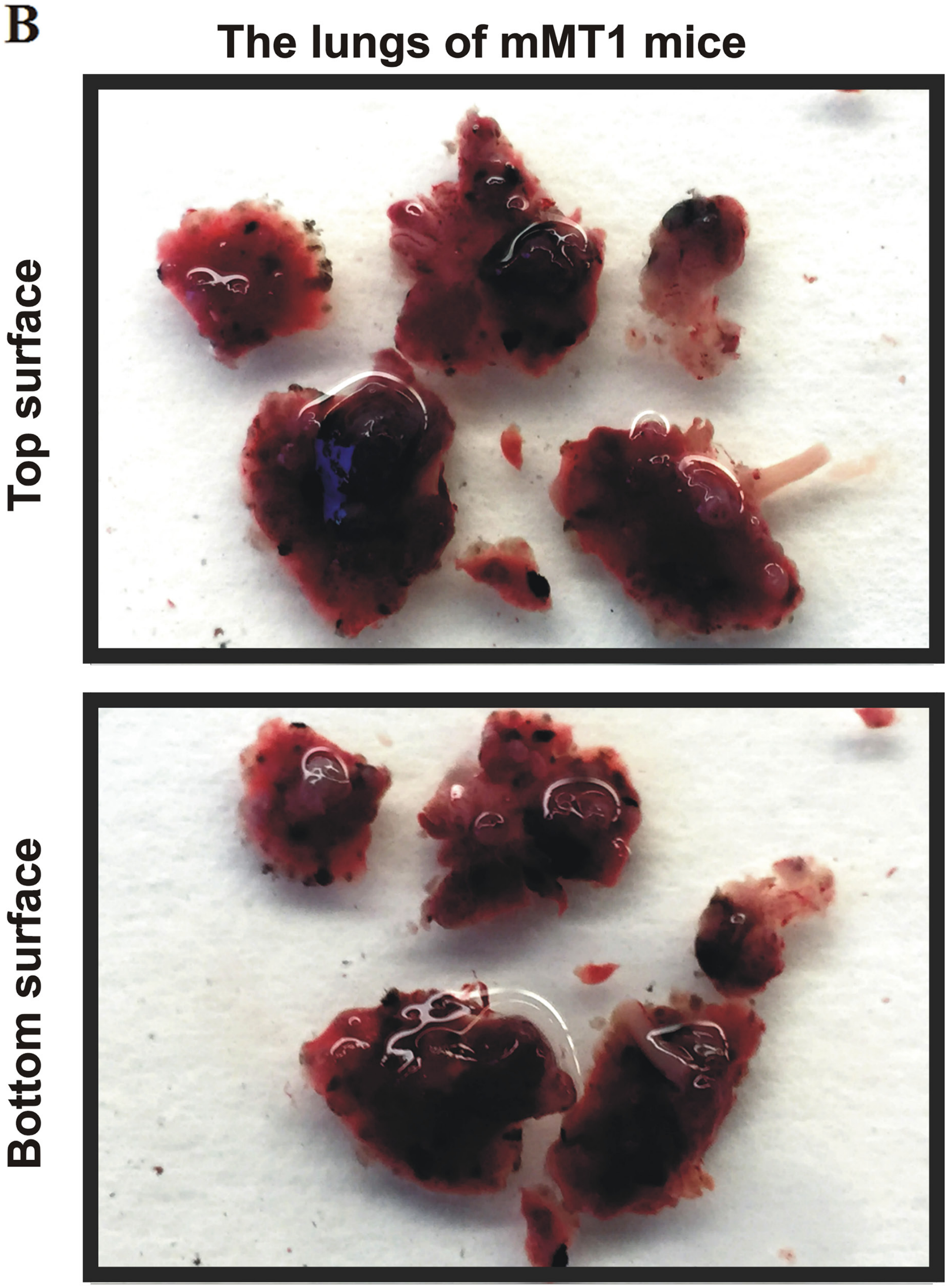

Supplementary Figure S2: (*Continued*) Metastatic nodules in mice. B. Pulmonary melanoma metastatic nodules were counted at day 23 post-cell injection in mice from the B16F1-mMT1 (mMT1) group, respectively. Representative images are shown. (*Continued*)

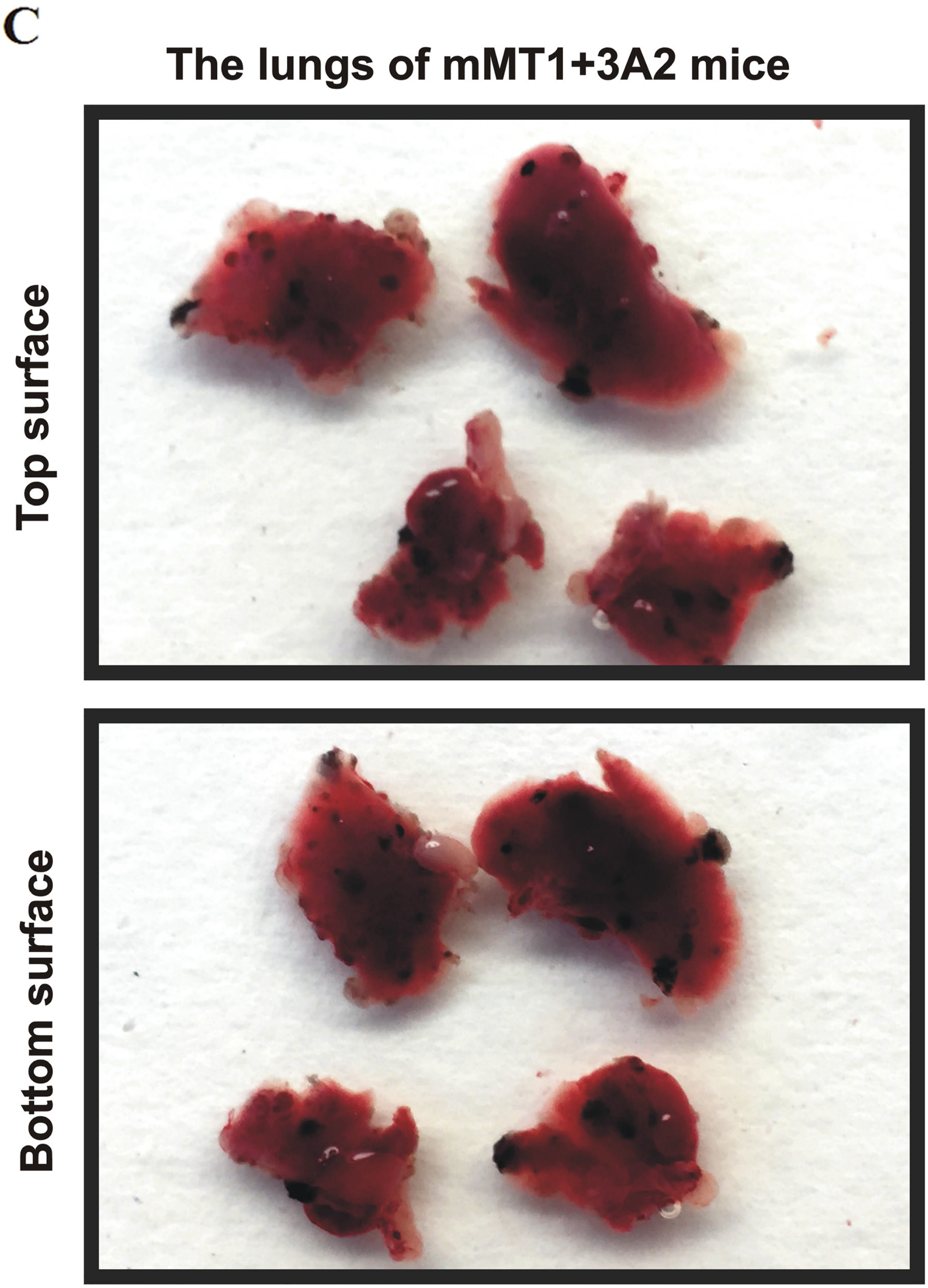

Supplementary Figure S2: (*Continued*) Metastatic nodules in mice. C. Pulmonary melanoma metastatic nodules were counted at day 23 post-cell injection in mice from the B16F1-mMT1+3A2 Fab (mMT1+3A2) group. Representative images are shown.

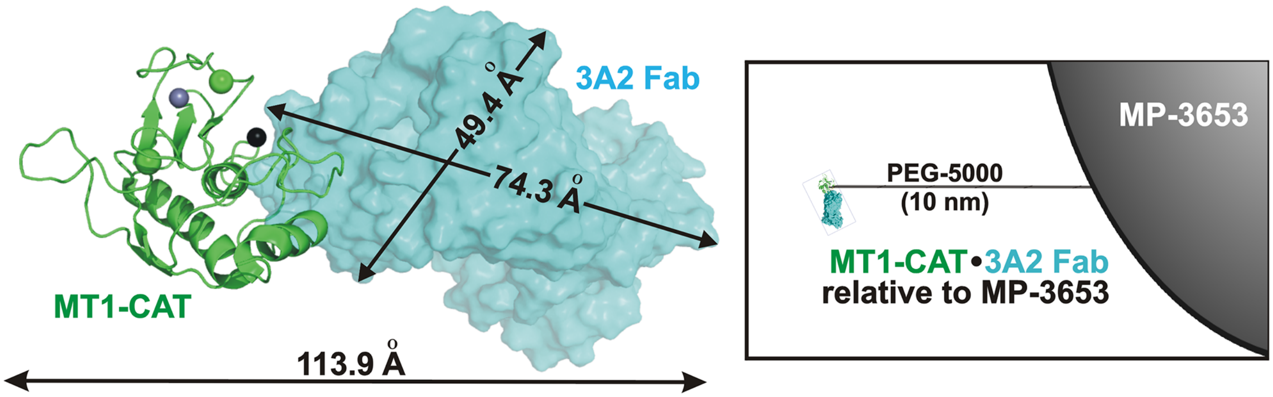

Supplementary Figure S3: The size of the 3A2 Fab *versus* the fluorescent imaging MP-3653 reporter. *Left*, The structure of MT1-CAT (*green*) in a complex with 3A2 Fab (*cyan*) (Figure 6A). The catalytic zinc, black sphere. The structural zinc and calcium, grey and green spheres, respectively. The width of the modeled 3A2 Fab (49.4 Å) was measured as the distance from the V_L Gly16 to the V_H Phe224. The length (74.3 Å) was determined as the distance between the V_L Lys190 to V_H Ala325. The distance between the outermost residues in the MT1-MMP•3A2 complex is 113.9 Å as measured between the 3A2 V_L Lys190 and the MT1-MMP Gly170. *Right*, a schematic representation (not to a scale) illustrates the size of the MT1-MMP•3A2 Fab complex relative to the 10 nm length of the PEG-5000 [51] in the structure of the fluorescent MP-3653 reporter with a hydroxamate warhead.
